# Unmet needs of patients with COPD in Germany: a retrospective, cross-sectional study

**DOI:** 10.1183/23120541.00976-2024

**Published:** 2025-06-16

**Authors:** Felix J.F. Herth, Claus F. Vogelmeier, Franziska C. Trudzinski, Henrik Watz, Dirk Skowasch, Kai-Michael Beeh, Chris Compton, Tharishini Mohan, Hartmut Richter, Jing Claussen, Sabine Bartel

**Affiliations:** 1 Thoraxklinik Heidelberg and Translational Lung Research Center Heidelberg, University of Heidelberg, Heidelberg, Germany; 2 Member of the German Center for Lung Research (DZL), Germany; 3 Universität Marburg, Marburg, Germany; 4 Velocity Clinical Research, Großhansdorf, Germany; 5 Lungenclinic Großhansdorf, Airway Research Center North, Großhansdorf, Germany; 6 Universitätsklinikum Bonn, Bonn, Germany; 7 Insaf Institut fuer Atemwegsforschung GmbH, Taunusstein, Germany; 8 Global Medical, GSK, London, UK; 9 Epidemiology, IQVIA, Frankfurt, Germany; 10 Medical Affairs, GSK, Munich Prinzregentenplatz, Germany

## Abstract

**Background:**

Earlier diagnosis and treatment of COPD, particularly preventing exacerbations, are key to slowing disease progression and reducing mortality. This study focused on the identification of patients in Germany with unstable COPD due to suboptimal treatments.

**Methods:**

The IQVIA™ LRx database, capturing 80% of Statutory Health Insurance prescriptions was used to identify patients with COPD using a machine-learning model. Patients with unstable COPD were identified through high prescriptions of oral corticosteroid (OCS) and/or rescue inhalers between April 2022 and March 2023.

**Results:**

The machine-learning model identified around 2.6 million treated patients with COPD, with 77% precision. The mean age was 71 years, 48% were female and 86% were aged ≥60 years. About 14% patients (n=363k) exhibited unstable COPD due to high OCS prescriptions, while 10% patients (n=256k) had high rescue inhaler prescriptions. Among those with high OCS and high rescue inhaler prescriptions, respectively, 43% and 38% were on dual therapy, 17% and 21% were on single inhaler triple therapy, 14% and 16% were on multiple inhaler triple therapy, 11% and 9% were on monotherapy and 15% and 17% had no maintenance therapy.

**Conclusions:**

A substantial number of unstable COPD patients were either on suboptimal maintenance therapy (monotherapy or inhaled corticosteroid-based dual therapy) or not receiving any maintenance therapy. The study highlights a substantial need in Germany for improved maintenance therapy, which could reduce disease burden, improve disease stability and reduce reliance on OCS and rescue therapies, thereby minimising side effects.

## Shareable abstract

**Many patients have unstable COPD and ineffective maintenance therapy in Germany. There is a substantial need for improved maintenance therapy to potentially reduce the burden of disease, improve stability, and reduce reliance on OCS and rescue therapies.** https://bit.ly/4i2dK9T

## Introduction

In Germany, 6.4% of the population aged over 40 years were diagnosed with COPD in 2017, showing regional variations [1]. Early diagnosis and treatment can have a significant public-health impact [2]. Recurrent episodes of exacerbations contribute to around 25% of the decline in lung function in patients with COPD [3]. Therefore, preventing exacerbations with appropriate maintenance therapy is vital to slow disease progression and lower mortality rates [4].

In Germany, both the German national guideline, Nationale Versorgungs Leitlinie (NVL) COPD 2021 and the Global Initiative for Chronic Obstructive Lung Disease (GOLD) 2024 recommendations are followed [5]. The NVL COPD 2021 guideline recommends using long-acting muscarinic antagonists (LAMAs) or long-acting β_2_-agonists (LABAs) as maintenance therapy for patients with mild-to-moderate symptoms. For those with moderate-to-severe symptoms, combining LAMA+LABA is recommended [6]. In case of exacerbations, escalation to LAMA+LABA is suggested for patients on LAMAs, while those on LAMA+LABA or LABA+ inhaled corticosteroid (ICS) and with increased eosinophil levels should consider escalation to the triple combination of LAMA+LABA+ICS [6]. According to the GOLD 2024 recommendations, initial treatment for symptomatic patients with ≥2 moderate exacerbations or ≥1 exacerbation leading to hospitalisation should be LABA+LAMA, as it is more effective than monotherapy [2]. Additionally, LABA+ICS treatment is not recommended as (initial) maintenance therapy in patients with COPD; instead, LABA+LAMA+ICS is preferred, wherein single inhaler combination therapies are recommended due to low adherence to multiple inhaler treatments [2, 7–9].

Despite guidelines, a substantial gap exists between recommended therapies and real-world practice, increasing the risk of exacerbations [10]. In Germany, triple-therapy use did not correlate with COPD exacerbation risk [11]. High prescriptions of oral corticosteroid (OCS) and rescue inhalers (*e.g.* short-acting β_2_-agonists (SABAs)) can be indicative of inadequate maintenance therapies, poor symptom control and frequent exacerbations [12, 13]. High OCS prescriptions reflect the extent of exacerbations in patients with COPD [13]. SABA reliever use predicts short- and long-term exacerbation risks in moderate-to-very-severe COPD patients with a history of exacerbations, emphasising its importance in assessing current symptom control and future risk [12].

This retrospective, cross-sectional study aims to focus on high OCS and SABA inhaler prescriptions as indicators of suboptimal disease control in patients with COPD in Germany. By leveraging a comprehensive prescription database, we seek to describe the population of patients with evidence of unstable COPD, who may have an unmet need due to suboptimal management.

## Methods

### Study design and aims

This study was conducted with data from April 2022 to March 2023, primarily aimed to evaluate and quantify patients with unstable COPD in Germany at a national level. Patients with unstable COPD were identified through high OCS prescriptions (>2 OCS prescriptions per year or >500 mg prednisone equivalents per year) and/or high prescriptions of rescue inhalers (SABA; ≥4 prescriptions per year). Secondary objectives included identifying the specialty treating these patients (pulmonologists *versus* general practitioners (GPs)) and assessing the frequency of visits to pulmonologists at least once per year.

### Data source and study population

The longitudinal prescription database IQVIA™ LRx was the primary data source, capturing approximately 80% of statutory health insurance (SHI) prescriptions from retail pharmacies in Germany. This anonymised database included details on prescribed product, substance, package form (identified by Pharmazentralnummer), prescription date, prescriber speciality, basic patient demographics (age and gender) and prescriber location. The dataset covered patients treated with core market drugs (supplementary table S1) during the study period, with prescriptions tracked back 2 years. Patients with >50 prescriptions/month were excluded to eliminate pseudo-patients, while retaining valid entries.

**FIGURE 1:**
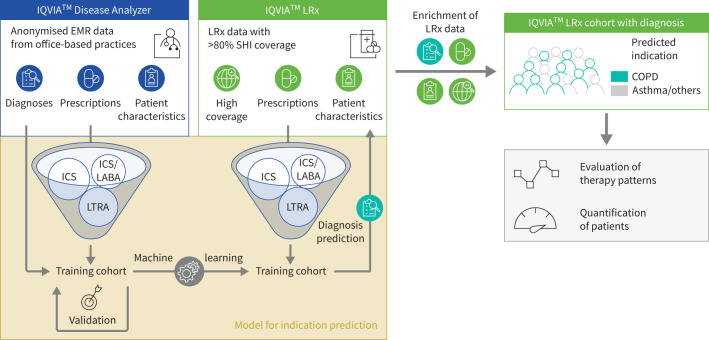
Prediction of diagnosis by a machine-learning model based on prescriptions. EMR: electronic medical record; ICS: inhaled corticosteroid; LABA: long-acting β_2_-agonist; LRx: Longitudinal Prescription database; LTRA: leukotriene receptor antagonist; SHI: statutory health insurance.

To address the lack of diagnosis data in IQVIA™ LRx, the IQVIA™ Disease Analyzer database was used to develop a machine-learning (ML) model for identifying patients with COPD (figure 1). The IQVIA^TM^ Disease Analyzer database included anonymised electronic medical records of patients who consulted GPs, pulmonologists and other specialities in Germany and had at least one SHI prescription of core market drugs (supplementary table S1). A training dataset from the IQVIA™ Disease Analyzer database (index period, October 2021 to September 2022) was used to construct an ML model for identification of patients with COPD in IQVIA™ LRx (figure 1), which had previously achieved overall accuracy of 89% for identifying patients with uncontrolled Global Initiative for Asthma step 4/5 asthma [14, 15]. After patient selection, diagnoses were tracked back 5 years and prescriptions for 2 years. Additional methodology details are in the online supplementary material. Data were calculated using absolute numbers and then rounded (to the nearest thousand) for reporting purposes, which may cause minor variations in totals. A single inhaler is indicated by a slash between medications (*e.g.* LAMA/LABA), while LAMA+LABA signifies the use of two separate inhalers.

## Results

### Identification of patients with COPD using a ML model

**FIGURE 2:**
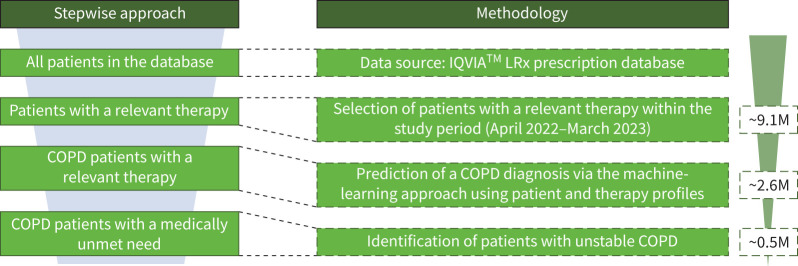
Identification of patients with COPD from IQVIA™ Longitudinal Prescription database (LRx). M: million.

In the IQVIA™ LRx prescription database, 6.4 million patients were identified as having received at least one relevant therapy prescription since 2020, with an estimated 9.1 million treated patients receiving at least one maintenance or reliever prescription during the analysis period (including all medications for obstructive pulmonary diseases, such as short-acting muscarinic antagonists, SABAs, LAMAs, LABAs and ICSs). This included patients with COPD, asthma and other respiratory conditions. A cohort of 313k patients from the IQVIA™ Disease Analyzer was used to develop the classification model, which achieved an overall accuracy of 71%. The model predictions are summarised in a confusion matrix (supplementary figure S1). The analysis projected 2.6 million treated patients with COPD (figure 2), with a precision of 77% for COPD, surpassing asthma (68%) and other respiratory disorders (69%). The recall for COPD was 67%, higher than other respiratory disorders (52%) but lower than asthma (83%). Over three-quarters of predicted COPD patients were correctly classified, capturing more than two-thirds of true-COPD patients (supplementary figure S1). Patients classified with both COPD and asthma were labelled as having COPD with an overlap of asthma or COPD/asthma.

### Baseline and clinical characteristics of patients with COPD

**FIGURE 3:**
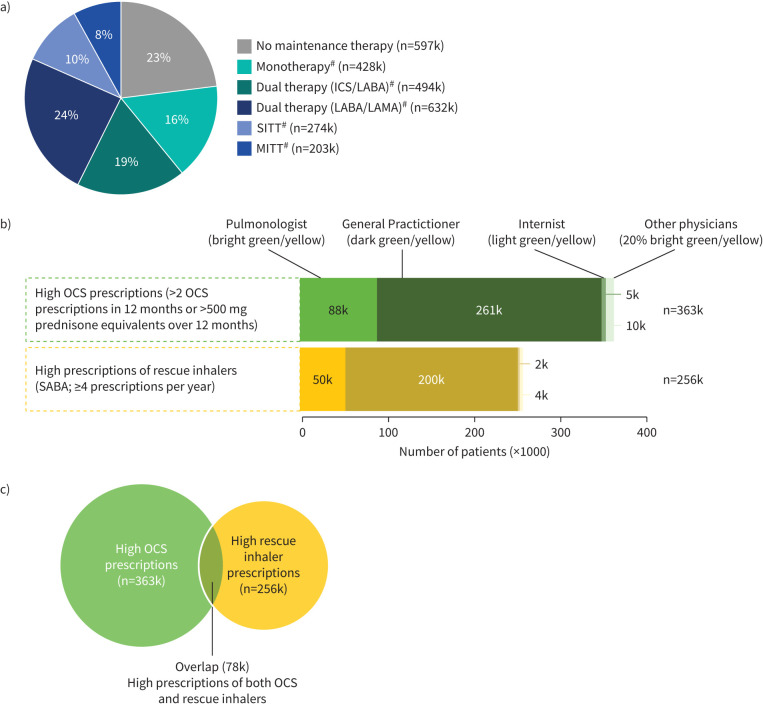
a) Patients with COPD by dominant maintenance therapy, b) patients with unstable COPD by high oral corticosteroid (OCS) use or high rescue inhaler use, c) patients with unstable COPD with both high OCS use and high rescue inhaler use. ICS: inhaled corticosteroid; k: thousand; LABA: long-acting β_2_-agonist; LAMA: long-acting muscarinic antagonist; MITT: multiple inhaler triple therapy; SABA: short-acting β_2_-agonist; SITT: single inhaler triple therapy. ^#^: monotherapy includes LABA or LAMA; ICS-based dual therapies include ICS/LABA, ICS+LABA or ICS+LAMA; dual therapy LABA/LAMA includes LABA/LAMA or LABA+LAMA; SITT includes ICS/LABA/LAMA; MITT includes IC+LABA+LAMA, ICS+LABA/LAMA or ICS/LABA+LAMA.

Demographic and clinical characteristics of patients with COPD are outlined in table 1**.** Among all patients with COPD (n=2628k), the mean age was 71 years, 48% (n=1251k) were female and the majority (86%, n=2256k) were aged ≥60 years. About 89% (n=2342k) had COPD only, while 11% (n=286k) had COPD/asthma. Approximately 30% (n=794k) of all COPD patients visited a pulmonologist, with increasing proportions among those with high OCS prescriptions (40%, n/n=144k/363k), high SABA prescriptions (44%, n/n=113k/256k) and both high OCS and SABA prescriptions (56%, n/n=44k/78k) (table 1). Dominant maintenance therapies among all patients with COPD were LABA/LAMA or LABA+LAMA (24%, n=632k), ICS+LABA, ICS/LABA or ICS+LAMA (19%, n=494k), monotherapies (LABA or LAMA; 16%, n=428k), single inhaler triple therapy (SITT) (ICS/LABA/LAMA; 10%, n=274k) and multiple inhaler triple therapy (MITT) (ICS+LABA+LAMA, ICS+LABA/LAMA or ICS/LABA+LAMA; 8%, n=203k). A substantial proportion of patients (23%, n=597k) had no maintenance therapy (table 1) (figure 3a). Antacids and antiflatulents (64%) and analgesics (60%) were top co-medications taken by patients with high OCS prescriptions (supplementary table S2).

**TABLE 1:** Patient demographics and clinical characteristics

Parameter	COPD only (n=2342k)	COPD/asthma (n=286k)	All COPD patients (n=2628k)	Pulmonologist visit (any) (n=794k)	No pulmonologist visit (n=1834k)
**Gender**					
Female	1100k (47)	151k (53)	1251k (48)	374k (47)	877k (48)
Age, years	71	71	71	69	72
**Age range, years**					
40–49	34k (1)	4k (1)	38k (1)	12k (2)	26k (1)
50–59	227k (10)	21k (7)	248k (9)	94k (12)	154k (8)
60–69	708k (30)	82k (29)	790k (30)	267k (34)	523k (29)
70–79	662k (28)	71k (25)	733k (28)	244k (31)	489k (27)
≥80	660k (28)	73k (26)	733k (28)	175k (22)	558k (30)
**Diagnosis**					
COPD only	2342k (100)	0	2342k (89)	734k (92)	1608k (88)
COPD/asthma	0	286k (100)	286k (11)	60k (8)	226k (12)
**Unstable COPD**					
High OCS prescriptions	336k (14)	27k (9)	363k (14)	144k (18)	218k (12)
High SABA prescriptions	243k (10)	13k (5)	256k (10)	113k (14)	143k (8)
High OCS and SABA prescriptions	76k (3)	2k (1)	78k (3)	44k (6)	34k (2)
**Maintenance therapy**					
No maintenance therapy	485k (21)	112k (39)	597k (23)	55k (7)	542k (30)
Monotherapy	418k(18)	9k (3)	428k (16)	107k (14)	320k (17)
ICS/LABA, ICS+LABA, ICS+LAMA	340k (15)	154k (54)	494k (19)	142k (18)	352k (19)
LABA/LAMA, LABA+LAMA	632k (27)	0.5k (0.2)	632k (24)	234k (29)	398k (22)
Triple therapy	467k (20)	10k (3)	477k (18)	256k (32)	221k (12)
MITT	197k (8)	6k (2)	203k (8)	113k (14)	91k (5)
SITT	270k (12)	4k (1)	274k (10)	143k (18)	131k (7)
**Pulmonologist visit**					
** **Yes	734k (31)	60k (21)	794k (30)	794k (100)	0

Data are presented as n (%) or mean. ICS: inhaled corticosteroid; LABA: long-acting β_2_-agonist; LAMA: long-acting muscarinic antagonist; MITT: multiple inhaler triple therapy; OCS: oral corticosteroid; SABA: short-acting β_2_-agonist; SITT: single inhaler triple therapy. k: thousand.

### Patients with unstable COPD

Among all patients with COPD, 14% (n=363k) exhibited unstable COPD characterised by high OCS prescriptions (>2 OCS prescriptions per year or >500 mg prednisone equivalents per year), 10% (n=256k) were characterised by high prescriptions of rescue inhalers (SABA, ≥4 prescriptions per year) (figure 3b), while 3% (n=78k) satisfied both the abovementioned categories (figure 3c). Among those with high OCS and high rescue inhaler prescriptions, respectively, 72% (n=261k) and 78% (n=200k) were primarily managed by GPs, 1% (n=5k) and 1% (n=2k) by internists, 24% (n=88k) and 19% (n=50k) by pulmonologists and 3% (n=10k) and 2% (n=4k) by other physicians (figure 3b).

#### High OCS prescriptions

**FIGURE 4:**
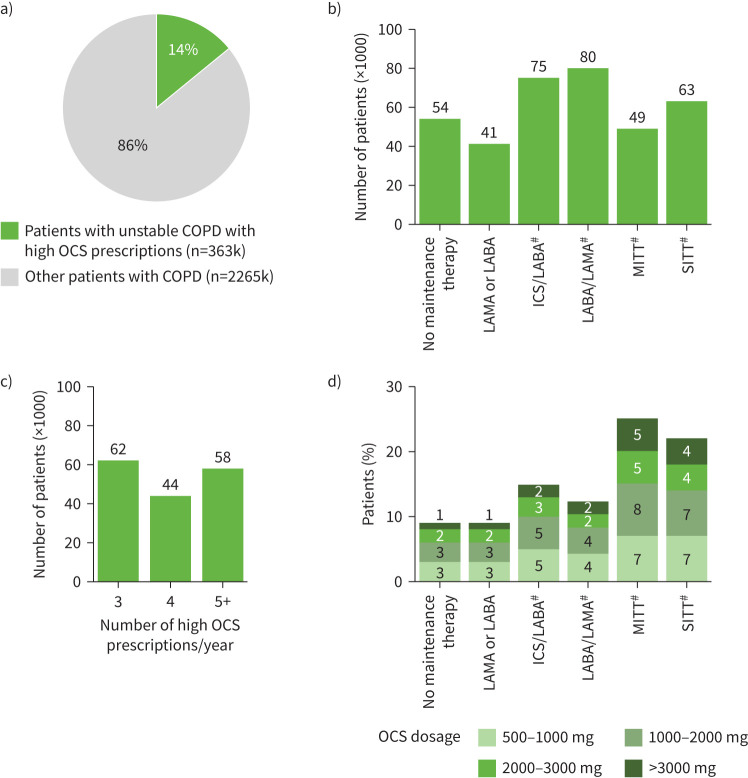
Treatment patterns in patients with unstable COPD with high oral corticosteroid (OCS) prescriptions. a) Proportion of patients with unstable COPD with high OCS prescriptions, b) dominant maintenance therapies of patients (×1000) with unstable COPD with high OCS prescriptions, c) number of patients with unstable COPD with high OCS prescriptions (>2 per year) and d) distribution of levels of high OCS prescriptions (>500 mg prednisone equivalents over 12 months) within each dominant COPD maintenance therapy. ICS: inhaled corticosteroid; k: thousand; LABA: long-acting β_2_-agonist; LAMA: long-acting muscarinic antagonist; MITT: multiple inhaler triple therapy; OCS: oral corticosteroid; SITT: single inhaler triple therapy. ^#^: ICS-based dual therapies include ICS/LABA, ICS+LABA or ICS+LAMA; dual therapy LABA/LAMA includes LABA/LAMA or LABA+LAMA; SITT includes ICS/LABA/LAMA; MITT includes ICS+LABA+LAMA, ICS+LABA/LAMA or ICS/LABA+LAMA.

Among all patients with COPD, 14% (n=363k) had unstable COPD, as indicated by receiving >2 OCS prescriptions per year or >500 mg prednisone equivalents per year (figure 4a). Among these, maintenance therapy distribution was as follows: 43% (n=156k) were on dual therapy (ICS+LABA, ICS+LAMA or ICS/LABA, n=75k; LABA+LAMA or LABA/LAMA, n=80k), 17% (n=63k) were on SITT (ICS/LABA/LAMA), 14% (n=49k) were on MITT (ICS+LABA+LAMA, ICS+LABA/LAMA or ICS/LABA+LAMA), and 11% (n=41k) on monotherapy (LAMA or LABA); while 15% (n=54k) lacked any maintenance therapy (figure 4b). Notably, about 23% (n/n=63k0/274k) of all patients with COPD on SITT maintenance therapy and about 24% (n/n=49k/203k) on MITT maintenance therapy exhibited high OCS prescriptions. Among all patients with COPD, around 6% (n=164k) were prescribed >2 OCS prescriptions per year (figure 4c). Of those on SITT, 7% of patients had OCS doses 1000–2000 mg, 4% had doses 2000–3000 mg and 4% had doses >3000 mg. Of those on MITT, 8% of patients had OCS doses 1000–2000 mg, 5% had doses 2000–3000 mg and 5% had doses >3000 mg (figure 4d).

#### High rescue inhaler prescriptions

**FIGURE 5:**
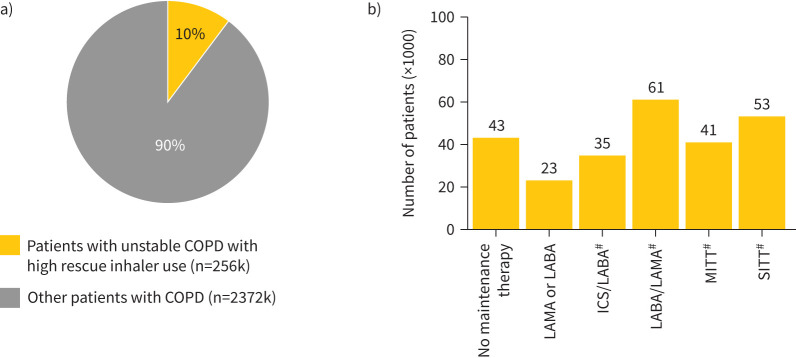
Treatment patterns in patients with unstable COPD with high rescue inhaler prescriptions. a) Proportion of patients with unstable COPD with high rescue inhaler use, b) dominant maintenance therapies of patients with unstable COPD with high rescue inhaler use (patients, ×1000). ICS: inhaled corticosteroid; k: thousand; LABA: long-acting β_2_-agonist; LAMA: long-acting muscarinic antagonist; MITT: multiple inhaler triple therapy; SITT: single inhaler triple therapy. ^#^: ICS-based dual therapies include ICS/LABA, ICS+LABA or ICS+LAMA; dual therapy LABA/LAMA includes LABA/LAMA or LABA+LAMA; SITT includes ICS/LABA/LAMA; MITT includes ICS+LABA+LAMA, ICS+LABA/LAMA or ICS/LABA+LAMA.

Among all patients with COPD, 10% (n=256k) had unstable COPD characterised by high rescue inhaler prescriptions (SABA; ≥4 prescriptions/year). Among these, 38% (n=96k) were on dual-maintenance therapy (ICS+LABA, ICS+LAMA or ICS/LABA, n=35k; LABA+LAMA or LABA/LAMA, n=61k), 21% (n=53k) on SITT, 16% (n=41k) on MITT and 9% (n=23k) on maintenance monotherapy (LAMA or LABA); while about 17% (n=43k) of patients lacked any maintenance therapy (figure 5). Notably, 19% patients (n/n=53k/274k) on SITT maintenance therapy (ICS/LABA/LAMA) and 20% (n/n=41k/203k) on MITT maintenance therapy (ICS+LABA+LAMA, ICS+LABA/LAMA or ICS/LABA+LAMA) exhibited high rescue inhaler prescriptions.

#### High prescriptions of both OCS and rescue inhalers

**FIGURE 6:**
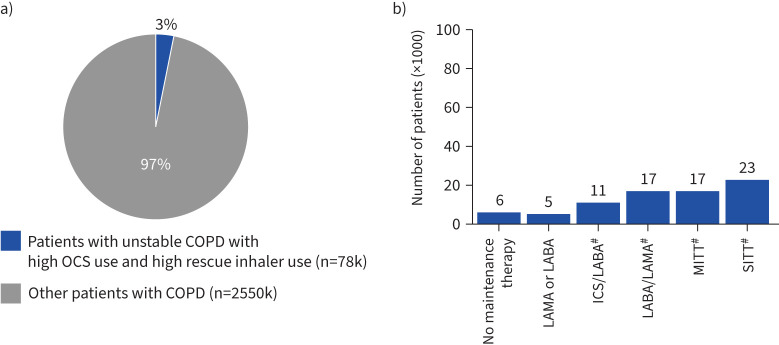
Treatment patterns in patients with unstable COPD with both high oral corticosteroid (OCS) prescriptions and high rescue inhaler prescriptions. a) Proportion of patients with unstable COPD with high rescue inhaler use, b) dominant maintenance therapies of patients (×1000) with unstable COPD with high rescue inhaler use. ICS: inhaled corticosteroid; k: thousand; LABA: long-acting β_2_-agonist; LAMA: long-acting muscarinic antagonist; MITT: multiple inhaler triple therapy; SITT: single inhaler triple therapy. ^#^: ICS-based dual therapies include ICS/LABA, ICS+LABA or ICS+LAMA; dual therapy LABA/LAMA includes LABA/LAMA or LABA+LAMA; SITT includes ICS/LABA/LAMA; MITT includes ICS+LABA+LAMA, ICS+LABA/LAMA or ICS/LABA+LAMA.

Among all patients with unstable COPD (n=541k), approximately 14% (n=78k) exhibited both high OCS and high rescue inhaler prescriptions, constituting about 3% of all patients with COPD (figure 6a). Among these, 35% (n=27k) were on dual-maintenance therapy (ICS+LABA, ICS+LAMA or ICS/LABA, n=11k; LABA+LAMA or LABA/LAMA, n=17k), 29% (n=23k) were on SITT, 22% (n=17k) were on MITT and 6% (n=5k) on maintenance monotherapy (LAMA or LABA); while about 7% (n=6k) of patients did not receive any maintenance therapy (figure 6b).

## Discussion

This study revealed that among the patients with COPD, about one in five had unstable COPD, characterised by high prescriptions of OCS and/or rescue inhalers. Of these patients with unstable COPD, nearly half were either on suboptimal maintenance therapy (such as monotherapy or ICS-based dual therapy) or were not receiving any maintenance therapy. By addressing this, it could potentially reduce the burden of disease for patients, improve disease stability and reduce reliance on OCS and rescue therapies, thereby minimising their associated side effects.

In this study, an estimated 2.6 million treated patients (3.7% of the German population aged ≥18 years in 2022) [16] were identified with COPD in the IQVIA™ LRx prescription database using the ML approach, achieving 71% accuracy while a previous accuracy level of 89% was reached for asthma [14]. The estimated prevalence rate for COPD is lower than published estimates (5.1%–11.6%) [1, 17, 18], likely due to factors such as patient selection (treated *versus* diagnosed population), year of analysis, assessment methodology (self-reported surveys *versus* claims databases) and differences in age groups [1, 17]. A substantial number may be undiagnosed, misdiagnosed or undertreated [19, 20]. Additionally, estimates based on respondents' self-assessments rather than self-reported medical diagnoses carry a higher risk of misclassifying patients with similar symptoms, such as asthma [17].

Among the 2.6 million treated patients with COPD, about one in five (21%) exhibited characteristics of unstable COPD indicated by high OCS and/or high rescue inhaler prescriptions. While this percentage might seem low, including untreated or undiagnosed patients could reveal substantial unmet needs. The NVL COPD 2021 guidelines advise against long-term OCS use [6], as prolonged OCS courses for COPD exacerbations are correlated with increased hospitalisations for pneumonia and all-cause mortality risks [21]. Higher OCS doses (>500 mg) elevate the risk of adverse outcomes [22]. Additionally, high usage of rescue inhalers such as SABAs reflects poor symptom control and is a marker of more severe and symptomatic disease [23]. Physicians should consider more intensive maintenance therapies for these patients, as high rescue medication use is associated with worse disease outcomes [23].

Nearly one in two patients (46%) with unstable COPD were on either suboptimal maintenance therapies or had no maintenance therapy at all. Suboptimal maintenance therapies, which do not adhere to GOLD guidelines, included monotherapies (11%; LAMAs or LABAs) and ICS-based dual therapies (18%; ICS+LABA, ICS+LAMA or ICS/LABA). Additionally, 17% patients received no maintenance therapy. This aligns with previous findings reporting treatment nonadherence to guidelines [10, 11], a substantial portion of patients with COPD not receiving any maintenance therapy [24] and under-utilisation of maintenance therapy in patients with COPD [25]. A considerable number of patients with unstable COPD could benefit from escalation to more appropriate therapies, such as dual or triple therapy, to reduce exacerbations [2], subsequently mitigating OCS overuse [26]. Our study revealed that even patients with unstable COPD, with potentially high exacerbation rates and poor symptom control, did not have escalation to appropriate maintenance therapy, revealing a gap in disease control and medication prescription. However, it remains unclear whether these patients would have received an escalation after a year of high OCS and SABA usage. Presumably, only a small percentage would have undergone such an escalation, as has been described in the other real-world study [27]. Appropriate maintenance therapy is crucial for reducing exacerbation rates and potentially lowering overall management costs for patients with COPD [28]. Our study, however, also identified a significant population of patients with COPD who seem to have poor disease control (high OCS and/or rescue inhaler prescriptions) but are already on inhaled triple therapy (31%), indicating the need for future precision medicine therapy options such as biologics [29].

Among those with high OCS and high rescue inhaler prescriptions, respectively, 72% and 78% were primarily managed by GPs, whereas only 24% and 19% were treated by pulmonologists. This discrepancy may be due to the scarcity of specialists in Germany [30], difficulty in obtaining appointments, especially in rural areas [31] and patient's attitudes and preferences [30]. Disparities exist among GPs and specialists regarding COPD diagnosis, treatment and practical implementation of education measures [32]. In Germany, specialist care can be sought without a GP referral, often resulting in GPs not receiving medical reports from specialists [33]. Despite the majority of Disease Management Programs (DMPs) being carried out by GP practices, a small percentage are exclusively managed by pulmonologists and other specialists [33]. Quality of care for patients with COPD in primary care networks has been reported to be suboptimal despite the presence of DMPs, likely reflecting broader challenges in German general practice [33]. Pulmonary specialists have been reported to prioritise the improvement of functional exercise capacity and quality of life (QoL), followed by prevention of exacerbations, aligning with national and international guidelines recommending OCS for exacerbations only [34]. Emphasising early diagnosis and appropriate treatment can prevent and alleviate symptoms, reduce exacerbation rates and severity, enhance exercise capacity and QoL and prolong survival [34].

A COSYCONET 2018 study examined adherence to prescribing guidelines for COPD in Germany and identified substantial deviations from the former GOLD guidelines, revealing undertreatment among patients in GOLD groups C and D with high exacerbation rates [5]. Our research analysed the high prescription rates of OCS and SABA, highlighting a considerable unmet need in COPD treatment in Germany. Addressing this requires optimising pharmacotherapy per guidelines, including escalating to triple therapy for exacerbations and closely monitoring patient progress [6]. Enhancing patient education, adherence to treatments and regular physician engagement is crucial [6]. We believe that establishing or strengthening robust regional healthcare networks could improve communication between GPs and pulmonologists, ensuring annual visits to pulmonologist and increasing awareness of exacerbations and OCS courses. Last, nonpharmacological interventions such as pulmonary rehabilitation, smoking cessation, physical activity and vaccinations should be emphasised to maintain or improve COPD stability [6, 35].

This study exhibits several key strengths. First, this study employed an IQVIA™ LRx prescription database containing 80% of statutory healthcare patients in Germany, facilitating analysis of a substantial patient cohort. Second, the study included a comprehensive analysis across Germany, unlike a previous study confined to North-Eastern Germany [36]. Furthermore, the study focused on patients treated by pulmonologists and/or GPs. Additionally, the analysis of ICS/LABA data is recent, aligning with the 2024 GOLD guidelines. Moreover, it enables OCS data examination from the whole of Germany, overcoming the limitations of claims analysis methods focusing on only North-Eastern Germany [36].

This study has several limitations. First, the analysis was restricted to statutory healthcare patients in the German dataset, excluding privately insured patients, about 10% of the population. Second, the analysis excluded patients treated for COPD or exacerbations in hospitals. Another limitation is the IQVIA™ LRx database's lack of diagnoses, necessitating an ML model for indication assignment. The ML model had a sensitivity of 77% in detecting patients with COPD, potentially causing slight overestimation or misclassification of the COPD patient population. Furthermore, the IQVIA™ LRx database does not record prescriptions outside its pharmacies and demographic data are restricted to age and often have unclear gender. Adherence to therapy is crucial for achieving optimal clinical outcomes in patients with COPD [37]. However, our study only assessed the fulfilment of prescriptions in the pharmacy and was unable to analyse whether the patients correctly adhered to their medications. The database covers about 80% of prescription claims from retail pharmacies reimbursed by SHI. To extrapolate to national totals, including patients with private insurance, a linear projection was used. The extrapolation factor was calculated based on the ratio of respiratory claims volumes in LRx compared with a complete census of SHI claims, along with a statistically validated sample of >6000 retail pharmacies reporting private sales. The analysis period coincided with the global coronavirus disease 2019 pandemic, possibly underestimating OCS use for exacerbations due to shielding measures, potentially preventing visits to doctors. Conversely, OCS may have been prescribed for reasons other than COPD exacerbations (*e.g.* due to other comorbidities). Our data do not account for OCS prescribed for home emergencies that patients might not use.

### Conclusions

Around one in five patients with COPD exhibited high OCS prescriptions and/or frequent prescriptions of rescue inhalers. Of these, nearly half were either on suboptimal maintenance therapy (such as monotherapy or ICS-based dual therapy) or were not receiving maintenance therapy at all. This study emphasises an unmet need in Germany regarding the optimisation of maintenance therapy for patients with unstable COPD, which could potentially improve disease stability, reduce the burden of disease for patients and reduce reliance on OCS and rescue therapies, thus minimising their side effects.

## Data Availability

The datasets used and/or analysed during the current study are available upon request from the corresponding author.

## Supplementary material

**Please note:** supplementary material is not edited by the Editorial Office, and is uploaded as it has been supplied by the author. Supplementary material 00976-2024.SUPPLEMENT
