# Supplementary material for: Unmet needs of patients with COPD in Germany: a retrospective, cross-sectional study
Source: ERJ Open Res. 2025 Jun 16;11(3):00976-2024. doi: 10.1183/23120541.00976-2024 (PMC12168169; doi:10.1183/23120541.00976-2024)
Supplement: Supplementary file 1 [file 00976-2024.SUPPLEMENT.pdf]

Supplementary material

Unmet needs in patients with COPD in Germany: a retrospective, cross-sectional study

Machine learning model

A training dataset, derived from the IQVIA™ Disease Analyzer database, was divided into training and validation subsets on a 3:1 basis, with the larger subset used for training and the smaller one for testing the model's accuracy (pre-index period). This part of the study was already finalised and optimised [1]. The same approach was already used to identify patients with uncontrolled severe asthma [2, 3]. The machine learning (ML) model for unstable chronic obstructive pulmonary disease (COPD) assessment and quantification was then applied to treated patients sourced from the longitudinal prescription database IQVIA™ LRx. Multiple model runs were conducted with varied random splits and the results aggregated to form a unified stable model. A gradient boosted tree model was trained using the IQVIA™ Disease Analyzer dataset to predict the indications in the IQVIA™ LRx dataset. Patients were categorised into their true indication, COPD or asthma, based on their history of World Health Organization International Classification of Diseases (ICD)-10 codes (supplementary table S1) documented in the physician practices within the preceding five years of the pre-index period. Patients lacking documented COPD or asthma diagnoses were categorised under the indication “Other respiratory conditions” encompassing relevant non-COPD and non-asthma conditions (supplementary table S1). A weighting scheme was implemented to assign a unique indication when multiple diagnoses were present: diagnoses directly linked to a prescription in the market were given the highest weight, followed by diagnoses issued on the same day as a market prescription. Diagnoses without a prescription and “Other respiratory conditions” diagnoses held lesser weight than COPD and asthma diagnoses, with decreasing weights for historical diagnoses.

The modelling process identified parameters most closely correlated with the true *a priori* classification in a dataset lacking diagnoses. To validate the model, a holdout sample not included in training was utilised, and predicted diagnoses were compared with the true indications assigned by ICD codes. Model results were evaluated based on (a) accuracy (the proportion of correct predictions among all data points), (b)

precision (the proportion of correct predictions for each class among all predictions for that class) and (c) recall (the proportion of correctly classified instances among all instances *a priori* assigned to that class) (supplementary table S3). Precision and recall were similarly calculated for asthma and other respiratory disorders. Overall precision and recall were calculated as macro-averaged parameters (simple average over the three class values) or as micro-averaged parameters (equivalent to accuracy). Given the descriptive nature of this secondary data study, sample size calculation and power analysis were not applicable, as no *a priori* hypothesis was tested.

Variables

The following variables, not directly included in the input data, were calculated, analysed and included in the study outcomes.

(a) Dominant maintenance therapy per patient was calculated based on prescriptions and therapy episodes within a 12-month time-period (supplementary table S4). All relevant prescriptions (supplementary table S1) being prescribed during or being partially consumed within the study time-period were considered. Therapy episodes were calculated based on prescriptions and corresponding durations. Each patient was assigned their dominant therapy, based on the highest number of days on therapy (total duration of episode) within the relevant timeframe.

(b) Dominant specialty: the dominant speciality was identified as the one with the highest prescription count during the study period. This analysis primarily focused on general practitioners, internal specialists, pulmonologists and outpatient centres. When determining the dominant speciality, all other specialities were treated as separate entities. However, once the analysis concluded, any patients assigned to a speciality other than the four mentioned were classified as “Other”.

(c) High prescriptions of oral corticosteroid (OCS): as a proxy for exacerbation, high prescriptions of OCS (Ephmra ATC: H02A2) were utilised, defined as >2 OCS prescriptions (each prescription day counting as one) within the study period, or >500 mg prednisone equivalents (supplementary table S5), calculated as the total mg prescribed during the study period.

(d) High short-acting β_2-agonist (SABA) prescriptions: to represent poor symptom control indicated by excessive use of emergency medications, high SABA prescriptions were assessed. High SABA prescriptions were determined by the number of SABA (Ephmra ATC: R03A4) or SABA/short-acting muscarinic antagonist (Ephmra ATC: R03L1) prescriptions (each prescription day counted as one) within the study period. High SABA prescriptions were defined as ≥4 prescriptions during the study period, under the assumption that frequent exacerbations constituted two or more exacerbations [4] and patients may stockpile rescue medication.

TABLE S1. Code lists

**Market definition of the core market (Ephmra ATC)**
R03A3 (LABA)
R03A4 (SABA)
R03B2 (xanthine)
R03D1 (ICS)
R03F1 (ICS/LABA)
R03H2 (PDE4i)
R03J2 (LTRA)
R03K1 (SAMA)
R03K2 (LAMA)
R03L1 (SABA/SAMA)
R03L2 (LABA/LAMA)
R03L3 (ICS/LABA/LAMA) Only if prescribed by specialists other than dermatologists and ENT-medicine
R03M0 (biologicals)
+ products XOLAIR, TEZSPIRE and DUPIXENT (biologicals)
**Maintenance therapy (Ephmra ATC)**
R03A3 (LABA)
R03D1 (ICS)
R03F1 (ICS/LABA)
R03K2 (LAMA)
R03L2 (LABA/LAMA)
R03L3 (ICS/LABA/LAMA)
**Indications (WHO ICD10 version 2019)**
Asthma: J45, J46
COPD: J41-J44
Other chronic respiratory: J31-J38, J47, J84, D86, E84.0, I27, M34.8
Other acute respiratory: J00-J18, J20-J22, J30, J36, J39, J40, J80-J82, J85, J86, J90, J91, J93-J96, J98, J99, A22.1, A70, B44.0, B96.0, B97.4, I26, I28, R05, R06, R09

ATC: anatomical therapeutic chemical; COPD: chronic obstructive pulmonary disease; ENT: ear, nose and throat; ICD: International Classification of Diseases; ICS: inhaled corticosteroid; LABA: long-acting β2-agonist; LAMA: long-acting muscarinic antagonist; LTRA: leukotriene receptor antagonist; PDE4i: phosphodiesterase-4 inhibitor; SABA: short-acting β2-agonist; SAMA: short-acting muscarinic antagonist; WHO: World Health Organization.

TABLE S2. Top 20 co-medications in patients with high OCS prescriptions per year

ATC2 code	Medication	Patients, n (%)
**H02**	Systemic corticosteroids	363k (100)
**A02**	Antacids and antiflatulents	233k (64)
**N02**	Analgesics	216k (60)
**C09**	Renin-angiotensin system antagonists	215k (59)
**J01**	Systemic antibacterials	213k (59)
**C03**	Diuretics	173k (48)
**C07**	β-blockers	173k (48)
**B01**	Antithrombotic agents	162k (45)
**M01**	Antirheumatic agents	149k (41)
**C10**	Lipid-regulating/anti-atheroma preparations	148k (41)
**C08**	Calcium channel blockers	99k (27)
**N06**	Psychoanaleptics	95k (26)
**H03**	Thyroid therapy	92k (25)
**N05**	Psycholeptics	84k (23)
**A10**	Antidiabetic drugs	83k (23)
**D07**	Topical corticosteroids	77k (21)
**G04**	Urologicals	63k (17)
**S01**	Ophthalmologicals	62k (17)
**N03**	Antiepileptics	60k (17)
**A11**	Vitamins	58k (16)

ATC: anatomical therapeutic chemical; OCS: oral corticosteroid.

TABLE S3. Matrix for computation of accuracy, precision and recall

		Predicted indication			
		Asthma	COPD	Other	Sum
**True indication**	Asthma	a	b	c	j (=a+b+c)
	COPD	d	e	f	k (=d+e+f)
	Other	g	h	i	l (=g+h+i)
	Sum	m (=a+d+g)	n (=b+e+h)	p (=c+f+i)	Total (T)

Accuracy=(a+e+i)/T; precision (COPD)=e/n; recall (COPD)=e/k.
COPD: chronic obstructive pulmonary disease.

TABLE S4. Classification of dominant therapy

Classification	Therapy classes
Monotherapy	ICS, LABA, LAMA
Dual therapy, fixed combination	ICS/LABA, LABA/LAMA
Dual therapy, free	ICS+LABA, ICS+LAMA, LABA+LAMA
Triple therapy, fixed combination	ICS/LABA/LAMA
Triple therapy, free	ICS+LABA+LAMA, ICS/LABA+LAMA, ICS+LABA/LAMA

ICS: inhaled corticosteroid; LABA: long-acting β_2-agonist; LAMA: long-acting muscarinic antagonist.

TABLE S5. Conversion factors for prednisone equivalent doses

Molecule	Factor
Prednisolone	1
Betamethasone	6.67
Dexamethasone	6.67
Methylprednisolone	1.25
Triamcinolone acetonide	1.25
Triamcinolone	1.25
Cloprednol	2
Cortisone	0.2
Hydrocortisone	0.25

FIGURE S1. **The confusion matrix for the AI/ML model distinguishes between asthma, COPD and other respiratory disorders among 313k patients (each having at least one prescription) whose data were extracted from the IQVIA™ Disease Analyzer**.

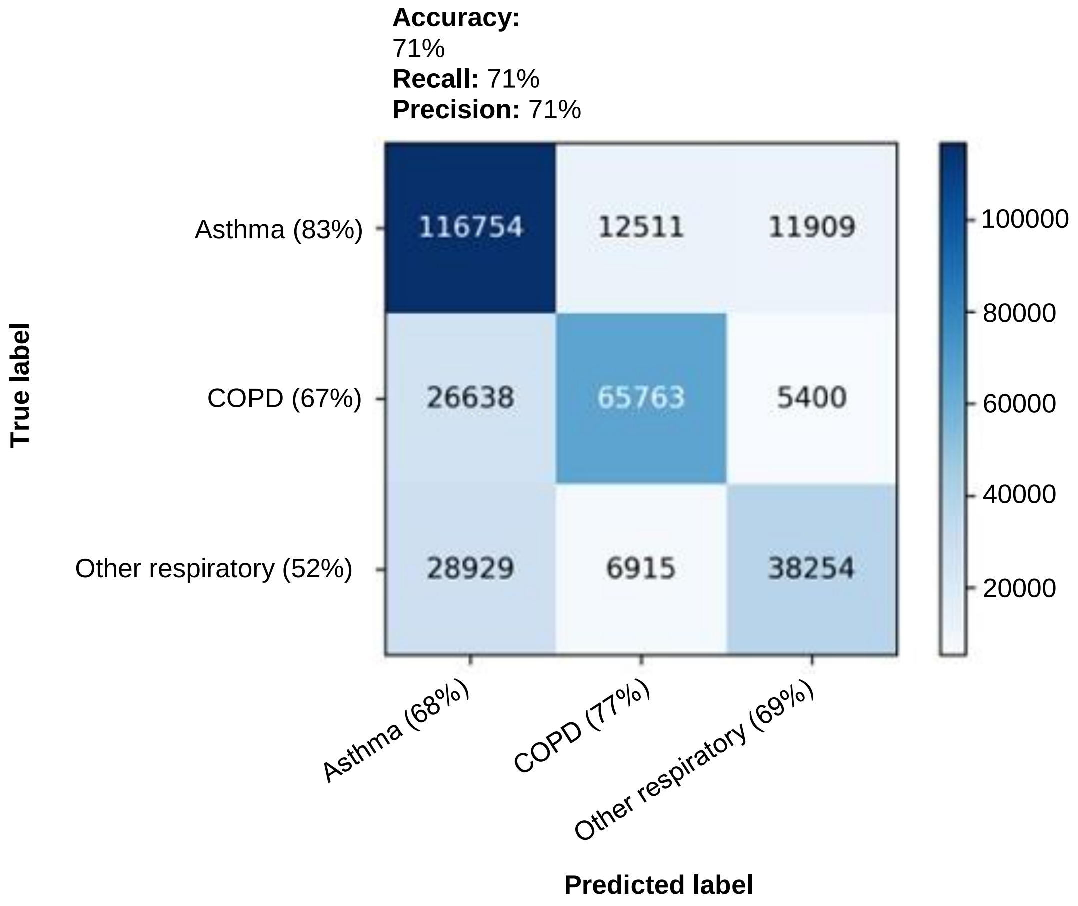

Accuracy, precision and recall are defined in supplementary table S3.

AI: artificial intelligence; COPD: chronic obstructive pulmonary disease; k, thousand; ML: machine learning.

Supplementary references

1 Rathmann W, Bongaerts B, Carius HJ, et al. Basic characteristics and representativeness of the German Disease Analyzer database. Int J Clin Pharmacol Ther 2018; 56: 459-466.

2 Bergmann KC, Skowasch D, Timmermann H, et al. Prevalence of patients with uncontrolled asthma despite NVL/GINA step 4/5 treatment in Germany. J Asthma Allergy 2022; 15: 897-906.

3 Timmermann H, Milger K, Virchow JC, et al. Health care situation in the treatment of uncontrolled GINA step 4/5 patients in Germany. J Asthma Allergy 2023; 16: 813-820.

4 Global Initiative for Chronic Obstructive Lung Disease. Global strategy for the diagnosis, management, and prevention of chronic obstructive pulmonary disease (2024 report). https://goldcopd.org/wp-content/uploads/2024/02/GOLD-2024_v1.2-11Jan24_WMV.pdf. Date last updated: 31 December 2023. Date last accessed: 6 May 2024.
